# 衍生化-超高效液相色谱-串联质谱法测定动物源性食品中维生素D和25-羟基维生素D

**DOI:** 10.3724/SP.J.1123.2024.02003

**Published:** 2025-03-08

**Authors:** Yu LIU, Jihui XIE, Pingping ZHANG, Di ZHOU, Weike ZHAO, Juzhou ZHANG

**Affiliations:** 安徽省食品药品检验研究院, 国家农副加工食品质量检验检测中心, 安徽 合肥 230051; Anhui Institute for Food and Drug Control, China National Center for Quality Supervision and Test of Agricultural-Avocation Processed Food, Hefei 230051, China

**Keywords:** 超高效液相色谱, 串联质谱, 维生素D, 25-羟基维生素D, 衍生化反应, 4-苯基-1,2,4-三唑啉-3,5-二酮, ultra performance liquid chromatography (UPLC), tandem mass spectrometry (MS/MS), vitamin D, 25-hydroxyvitamin D, derivatization, 4-phenyl-1,2,4-triazoline-3,5-dione (PTAD)

## Abstract

动物源性食品是人体摄入维生素D和25-羟基维生素D的重要食物来源。目前我国相关标准限于维生素D的含量测定,使用非衍生化方法进行检测。本研究以4-苯基-1,2,4-三唑啉-3,5-二酮(PTAD)作为衍生试剂,通过Diels-Alder反应,在维生素D和25-羟基维生素D上引入易电离基团,提高离子化效率,建立了衍生化-超高效液相色谱-串联质谱法(UPLC-MS/MS)同时测定动物源性食品中维生素D和25-羟基维生素D的检测方法。本研究对衍生化条件、样品前处理条件、色谱分离条件和质谱检测条件进行了优化。结果表明,在乙腈溶剂中,目标化合物与衍生试剂PTAD质量比为1∶10000的条件下,衍生反应充分;衍生反应1 h后达到稳定;相较于Silica和C_18_固相萃取(SPE)柱,HLB SPE柱对目标化合物的回收率更高,同时可有效降低基质效应的影响;在水-甲醇流动相体系中添加5 mmol/L甲胺,可显著提升目标化合物的检测灵敏度。样品经过均质,加入同位素内标,碱液皂化,萃取浓缩,固相萃取柱净化和化学衍生,在Waters Acquity UPLC BEH C_18_色谱柱(100 mm×2.1 mm, 1.7 μm)上,以0.1%甲酸-5 mmol/L甲胺水溶液和0.1%甲酸-5 mmol/L甲胺甲醇溶液作为流动相梯度洗脱。分析物在电喷雾正离子模式(ESI^+^)下进行多反应监测(MRM)测定,内标法定量。结果表明,维生素D和25-羟基维生素D在0.2~50 μg/L内线性良好,相关系数为0.9995~0.9999,方法检出限为0.018~0.066 μg/kg,方法定量限为0.06~0.22 μg/kg,在0.20、1.0、5.0 μg/kg 3个加标水平下,加标回收率为92.6%~99.4%,相对标准偏差为3.6%~6.2%。该方法灵敏度高,重复性好,适用于动物源性食品中维生素D和25-羟基维生素D灵敏、准确的定量检测。

## Abstract (en)

Animal-derived foods are essential sources of vitamin D and 25-hydroxyvitamin D in the human diet. Currently, the relevant regulatory methods in China are limited to using non-derivatization methods to determine vitamin D content. In this study, 4-phenyl-1,2,4-triazoline-3-5-dione (PTAD) was used as a derivative reagent, and the ionization efficiencies of vitamin D and 25-hydroxyvitamin D were improved by introducing readily ionizable groups via the Diels-Alder reaction. This method allowed for the simultaneous determination of vitamin D and 25-hydroxyvitamin D in animal-derived foods using derivatization and ultra performance liquid chromatography-tandem mass spectrometry (UPLC-MS/MS). The conditions for derivatization, sample pretreatment, chromatographic separation, and MS detection were optimized. The results showed that the derivatization reaction was effective in acetonitrile solvent at a target compound to PTAD mass ratio of 1∶10000 and stabilized after 1 h. Compared with Silica and C_18_ solid-phase extraction (SPE) columns, hydrophilic lipophilic balanced (HLB) SPE columns yielded higher recoveries of the target compounds, while simultaneously reducing the matrix effect. The detection sensitivity was significantly improved by adding 5 mmol/L methylamine to the water-methanol mobile phase system. An isotopic internal standard was added to the homogenized samples, which were saponified with alkali solution, extracted and concentrated, purified using a SPE column, derivatized, and separated on a Waters Acquity UPLC BEH C_18_ column (100 mm×2.1 mm, 1.7 μm) with a gradient elution using 0.1% formic acid-5 mmol/L methylamine aqueous solution and 0.1% formic acid-5 mmol/L methylamine of methanol as the mobile phases. The analytes were determined by multiple reaction monitoring (MRM) in positive electrospray ionization mode (ESI^+^) and quantified using the internal standard method. The results for both vitamin D and 25-hydroxyvitamin D showed good linearity in the range of 0.2-50 μg/L, with correlation coefficients of 0.9995-0.9999. The limits of detection (LODs) and limits of quantification (LOQs) were in the range of 0.018-0.066 and 0.06-0.22 μg/kg, respectively. Recoveries were 92.6%-99.4% at three spiked levels (0.20, 1.0, and 5.0 μg/kg), and the relative standard deviations (RSDs) were 3.6%-6.2%. This highly sensitive method yields reproducible and accurate results for the quantitative determination of vitamin D and 25-hydroxyvitamin D in animal-derived foods.

维生素D以维生素D_2_(麦角钙化醇,VD_2_)和维生素D_3_(胆钙化醇,VD_3_)两种形式存在,都是具有甾体结构的脂溶性维生素。VD_2_主要由食用菌类或酵母合成,通过紫外线辐射作用将麦角固醇转化为VD_2_^[1,2]^。人体中VD_3_主要由7-脱氢胆固醇通过紫外线辐射转化合成,或者通过摄入动物源性食品获得^[3]^。由膳食摄入和皮肤合成两种途径获得的维生素D通过血液运输到肝脏,产生以25-羟基维生素D_2_(25(OH)D_2_)和25-羟基维生素D_3_(25(OH)D_3_)两种形式存在的稳定代谢物。在肾脏中,代谢物被进一步羟基化为稳定性较差的1,25-二羟基维生素D,其生物活性达到最高^[4]^。维生素D的主要生理功能是促进肠道黏膜对钙和磷的吸收。人体中维生素D缺乏会影响骨骼健康,引起佝偻病、软骨病和骨质疏松等^[5,6]^。近年来的研究发现,维生素D还具有其他生物效应,如调节激素分泌、影响免疫功能、调节细胞增生与分化,其与多种疾病的发生发展密切相关^[7,8]^。

维生素D与25-羟基维生素D的天然食物来源主要为动物源性食品,例如鱼类、肉类、鸡蛋以及乳制品等^[9]^。由于食品基质中维生素D及25-羟基维生素D的含量较低,基质干扰比较严重,通常采用碱皂化方法去除油脂等干扰物质,再进行萃取和净化处理^[10⇓⇓⇓⇓-15]^。在GB 5009.296-2023《食品安全国家标准 食品中维生素D的测定》^[16]^中,维生素D的检测方法有正相色谱净化-反相液相色谱法、在线柱切换-反相液相色谱法和液相色谱-串联质谱法(LC-MS/MS),其中液相色谱法中样品前处理过程需要使用正相半制备高效液相色谱或者使用一维液相色谱通过C_8_柱净化,步骤复杂,灵敏度不高,无法满足动物源性食品中微痕量组分的检测要求。在生物医学领域,有关25-羟基维生素D检测方法的研究较多,主要有免疫分析法和LC-MS/MS^[17⇓⇓-20]^。免疫分析法包括放射免疫法、酶联免疫吸附法和化学发光免疫法等^[21,22]^。免疫分析法操作相对简单,前处理不需要预纯化,缺点是不能区分不同形式的维生素D,测定结果为维生素D的总量;酶促反应易受温度、时间和抗体状态等因素影响;免疫分析法对基质敏感,容易与其他非目标物质产生交叉反应,特异性较差^[23]^。

GB 5009.296-2023方法限于维生素D的含量测定,其中第三法LC-MS/MS采用非衍生化方法检测,维生素D分子结构中没有质子化位点,较难在溶液中形成质子化的离子,在电喷雾电离模式下的检测灵敏度较低^[16,24,25]^。维生素D和25-羟基维生素D均含有共轭双烯结构,可与衍生试剂4-苯基-1,2,4-三唑啉-3,5-二酮(PTAD)发生反应(图1)。本实验基于Diels-Alder衍生化反应,使目标化合物与PTAD形成高相对分子质量、易电离的化合物,在电喷雾正离子模式下容易形成加氢峰[M+H]^+^,提高电离效率。衍生物的相对分子质量转移至较高质量范围,从而降低了背景噪声的干扰。

**图1:**
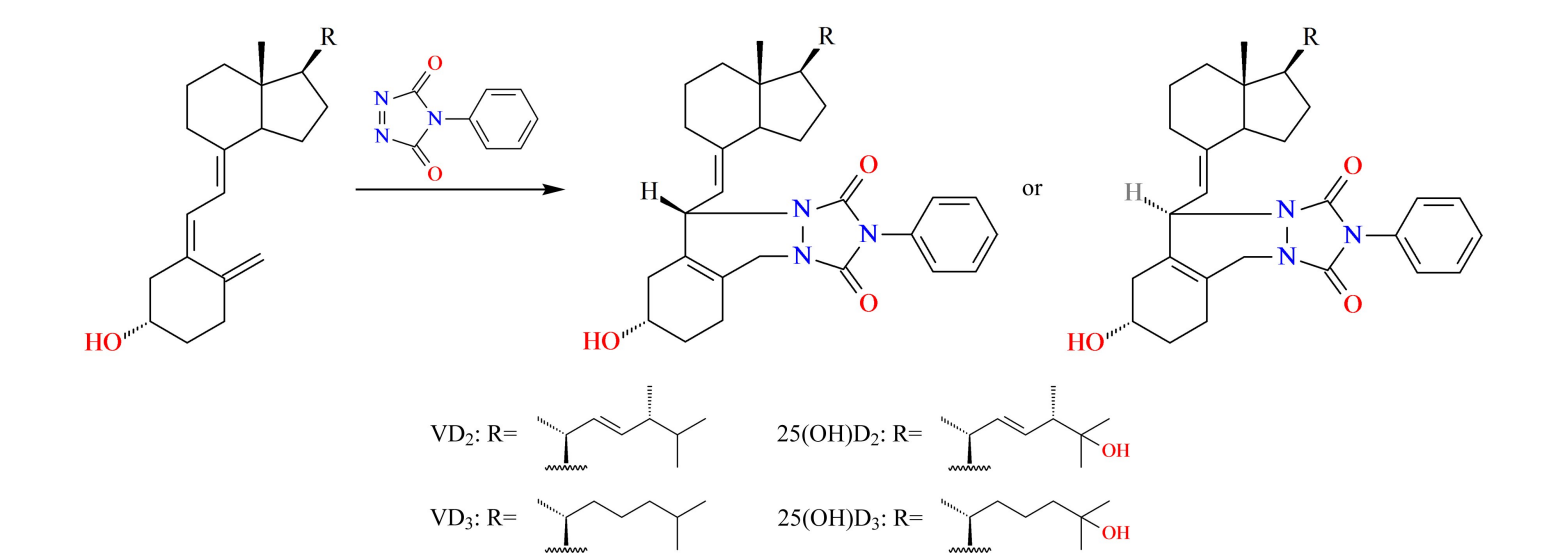
维生素D及25-羟基维生素D与PTAD的衍生反应

目前,衍生化-液相色谱-串联质谱同时检测动物源性食品中维生素D和25-羟基维生素D含量的研究较少^[3,5]^。本实验使用PTAD作为衍生试剂,建立了一种具有高特异性和灵敏度的衍生化-液相色谱-串联质谱同时测定维生素D和25-羟基维生素D的检测方法,为动物源性食品中维生素D和25-羟基维生素D的定性定量检测、营养含量分析和监管提供了技术支撑。

## 1 实验部分

### 1.1 仪器、试剂与材料

EXPEC 5210超高效液相色谱-三重四极杆质谱联用仪(杭州谱育科技公司); AutoVap S60型多样品自动氮吹仪(美国ATR公司); Smart E6 Plus全自动液液萃取仪(济南阿尔瓦仪器公司); R-210旋转蒸发仪(瑞士步琦公司); SHA-C水浴恒温振荡器(常州丹瑞仪器公司); 20位固相萃取装置(美国Agilent公司); Milli-Q超纯水制备仪(美国Millipore公司)。

25-羟基维生素D_2_(CAS号:21343-40-8)、25-羟基维生素D_2_-d_6_(CAS号:1262843-46-8)、25-羟基维生素D_3_(CAS号:19356-17-3)、25-羟基维生素D_3_-d_6_(CAS号:78782-98-6)、维生素D_2_(CAS号:50-14-6)、维生素D_2_-d_3_(CAS号:1217448-46-8)、维生素D_3_(CAS号:67-97-0)、维生素D_3_-d_3_(CAS号:80666-48-4)标准品均购自美国Sigma公司(质量浓度均为100 mg/L);Oasis HLB(200 mg/6 mL)、Sep-Pak Silica(500 mg/6 mL)、Sep-Pak C_18_(500 mg/6 mL)固相萃取柱均购自美国Waters公司;乙酸乙酯、正己烷、乙腈、甲醇和甲胺(色谱纯,德国Merck公司); PTAD(纯度97%,美国Sigma公司);甲酸(FA,纯度>99%,美国Fisher公司);石油醚、氢氧化钾、无水硫酸钠、抗坏血酸、2,6-二叔丁基对甲酚(BHT)和无水乙醇均为分析纯。

实验用鱼肉、虾肉、猪肉、羊肉、鸡蛋、鸭血和脱脂乳粉购自合肥市超市。

### 1.2 溶液的配制

空白基质溶液:取脱脂乳粉5.0 g,按1.3.1~1.3.3节步骤进行前处理(不加入混合内标使用液),用1.0 mL甲醇溶解。

标准使用液:取10 μL标准品溶液加入990 μL空白基质溶液后混匀,取10 μL混匀溶液加入990 μL空白基质溶液,混匀后制得质量浓度为0.01 mg/L的标准使用液。

混合标准使用液:分别取标准品溶液各100 μL,置于10 mL棕色容量瓶中,用甲醇定容,混匀,得到质量浓度为1.0 mg/L的混合标准使用液。

混合内标使用液:分别取同位素内标溶液各100 μL,置于10 mL棕色容量瓶中,用甲醇定容,混匀,得到质量浓度为1.0 mg/L的混合内标使用液。

标准工作溶液:分别吸取混合标准使用液0.02、0.05、0.1、0.5、1.0、5.0 mL,置于10 mL棕色容量瓶中,各加入混合内标使用液0.5 mL,用甲醇定容,混匀。分别吸取上述标准系列溶液各100 μL,置于离心管中,氮吹至干,再分别加入含有1.0 g/L PTAD的乙腈溶液1 mL,配制得到0.2、0.5、1.0、5.0、10和50 μg/L的系列标准工作溶液。

### 1.3 样品前处理

#### 1.3.1 皂化

称取2~5 g(精确至0.01 g)经均质处理的固体试样或50 g(精确至0.01 g)液体样品于250 mL平底烧瓶中,固体试样加入50 mL温水(40~45 ℃),用甲醇将混合内标使用液稀释至50 μg/L,吸取100 μL加入平底烧瓶中,再加入1.0 g抗坏血酸和0.1 g BHT,混匀。加入100 mL无水乙醇,加入20 mL氢氧化钾溶液(50%,质量分数),边加边振摇,混匀后于恒温振荡器中皂化,温度(80±2) ℃,时间30 min,皂化后立即用冷水冷却至室温。

#### 1.3.2 提取

将皂化液用30 mL水转入500 mL的分液漏斗中,加入100 mL石油醚后使用液液萃取仪振荡萃取5 min,取下层溶液再次萃取,合并醚层,用水洗至中性。将洗涤后的醚层过无水硫酸钠粉末填充的玻璃层析柱,滤入250 mL旋转蒸发瓶中,用15 mL石油醚冲洗后合并,水浴中减压旋蒸,待瓶中醚液剩下约2 mL时,取下蒸发瓶,氮吹至干。

#### 1.3.3 净化

用甲醇-水(9∶1, v/v)分次将蒸发瓶中的残留物溶解并转移至5 mL容量瓶中,定容待净化。将Oasis HLB固相萃取柱依次用6 mL乙酸乙酯、6 mL甲醇、6 mL水进行活化,取待净化样品过柱,用6 mL水、6 mL甲醇-水(7∶3, v/v)淋洗,用真空泵抽干固相萃取柱,用乙酸乙酯6 mL洗脱,使用氮吹仪吹干。

#### 1.3.4 衍生

加入1.0 mL含有1.0 g/L PTAD的乙腈溶液,涡旋10 s,室温下衍生1 h后,过0.22 μm有机系滤膜后供LC-MS/MS分析。

### 1.4 分析条件

色谱条件:Waters Acquity UPLC BEH C_18_色谱柱(100 mm×2.1 mm, 1.7 μm);流动相A为0.1%甲酸-5 mmol/L甲胺水溶液,流动相B为0.1%甲酸-5 mmol/L甲胺甲醇溶液,流速为0.35 mL/min,柱温40 ℃,进样量为10 μL。梯度洗脱:0~1.0 min, 60%B; 1.0~10.0 min, 60%B~100%B; 10.0~15.0 min, 100%B; 15.0~15.5 min, 100%B~60%B; 15.5~18 min, 60%B。

质谱条件:电喷雾电离(ESI)源,正离子模式;干燥气温度:270 ℃;干燥气流量:10 L/min;雾化器压力:345 kPa(50 psi);鞘气温度:400 ℃;鞘气流量:11 L/min;毛细管电压:4000 V;喷嘴电压:500 V;检测方式:多反应监测(MRM)模式;保留时间、加合离子、定性离子对、定量离子对及碰撞能量等参数见表1。

**表1:** 维生素D、25-羟基维生素D及其同位素内标衍生产物的质谱参数

Compound	t_R_/min	M_r_	Adduct ion	Precursor ion (m/z)	Product ions (m/z)	CEs/eV	Fragment voltage/V
25(OH)D_2_-PTAD	6.68	588.3	[M+CH_3_NH_3_]^+^	619.4	280.25, 298.15^*^	28, 14	155
25(OH)D_2_-d_6_-PTAD	6.66	594.3	[M+CH_3_NH_3_]^+^	625.4	280.25, 298.15^*^	28, 14	155
25(OH)D_3_-PTAD	6.57	576.3	[M+CH_3_NH_3_]^+^	607.4	280.25, 298.15^*^	28, 10	125
25(OH)D_3_-d_6_-PTAD	6.55	582.3	[M+CH_3_NH_3_]^+^	613.4	280.25, 298.15^*^	28, 10	125
VD_2_-PTAD	8.93	572.3	[M+CH_3_NH_3_]^+^	603.4	280.15, 298.15^*^	16, 17	155
VD_2_-d_3_-PTAD	8.91	575.3	[M+CH_3_NH_3_]^+^	606.4	283.15, 301.18^*^	16, 17	155
VD_3_-PTAD	8.96	560.3	[M+CH_3_NH_3_]^+^	591.4	280.15, 298.15^*^	20, 20	155
VD_3_-d_3_-PTAD	8.94	563.3	[M+CH_3_NH_3_]^+^	594.4	283.15, 301.18^*^	20, 20	155

*M*_r_: molecular mass of the derivatives; * quantitative ions; CEs: collision energies.

## 2 结果与讨论

### 2.1 衍生溶剂及浓度的优化

衍生化反应中,一般要求衍生试剂过量且稳定,与目标化合物反应完全。本研究考察了在不同溶剂环境下,衍生试剂PTAD的添加量对维生素D及25-羟基维生素D衍生化反应的影响。使用甲醇、乙酸乙酯和乙腈分别配制不同质量浓度的PTAD溶液,质量浓度分别为1.0、5.0、10、50、100和1000 mg/L。分别取1.2节中1 mL标准使用液于不同离心管中,氮吹至干,各加入1 mL上述不同质量浓度的PTAD溶液,涡旋10 s,室温下反应1 h,进行LC-MS/MS分析。通过衍生产物峰面积分析目标化合物衍生反应的转化率,结果见图2。结果表明,在甲醇溶剂中,衍生产物得率最低。在乙酸乙酯和乙腈溶剂中,当目标化合物与衍生试剂PTAD质量比为1∶10000时,衍生反应最充分。在PTAD质量浓度较低情况下,相较于其他溶剂,乙腈溶剂中衍生化产物得率最高。因此本试验选择乙腈为反应溶剂,目标化合物与衍生试剂PTAD质量比为1∶10000,此条件下衍生反应充分。

**图2:**
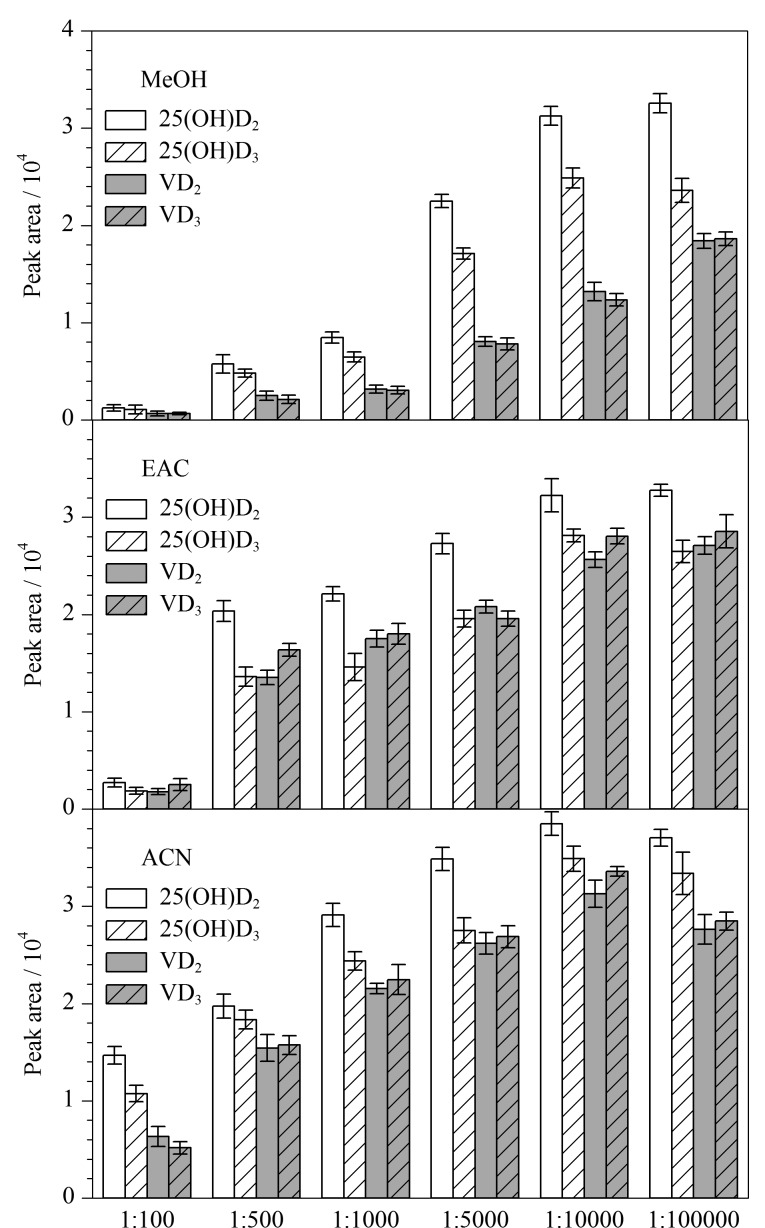
不同溶剂中维生素D及25-羟基维生素D与PTAD 的质量比对衍生产物峰面积的影响(*n*=3)

### 2.2 衍生时间的优化

分别取1 mL标准使用液于不同离心管中,氮吹至干,各加入1 mL质量浓度为100 mg/L的PTAD乙腈溶液,涡旋10 s,室温下反应,在不同时间点进行LC-MS/MS分析,结果见图3。结果表明,衍生反应在1 h时达到稳定状态,衍生产物在24 h内保持稳定。

**图3:**
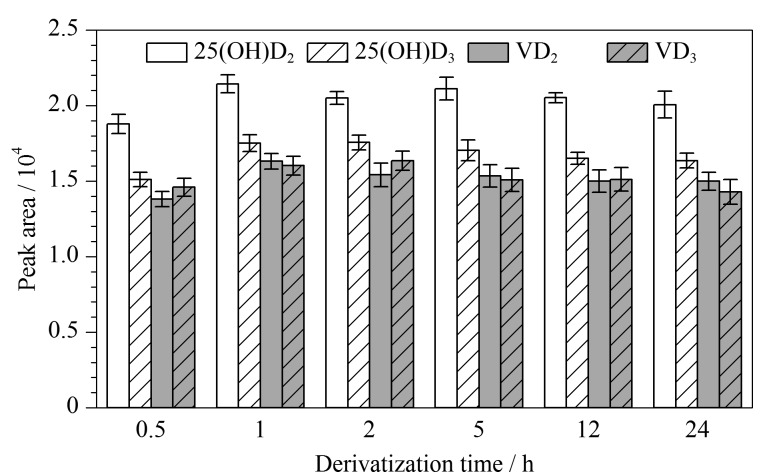
维生素D及25-羟基维生素D与PTAD衍生时间对衍生产物峰面积的影响(*n*=3)

### 2.3 固相萃取条件的优化

本研究比较了HLB、Silica和C_18_固相萃取柱对维生素D及25-羟基维生素D的回收率。分别配制含10 ng目标化合物的混合标准溶液,氮吹至干,溶解后过不同SPE柱。处理过程如下,HLB SPE柱:甲醇-水(9∶1, v/v)溶解,过预先活化好的HLB柱,依次用6 mL水、6 mL甲醇-水(7∶3, v/v)淋洗,6 mL乙酸乙酯洗脱;Silica SPE柱:正己烷溶解,过预先活化好的Silica柱,用6 mL乙酸乙酯-正己烷溶液(5∶95, v/v)淋洗,6 mL乙酸乙酯洗脱;C_18_ SPE柱:甲醇-水(9∶1, v/v)溶解,过预先活化好的C_18_柱,6 mL甲醇-水(7∶3, v/v)淋洗,6 mL甲醇洗脱。分别收集洗脱液,氮吹至干,加入1.0 mL含有1.0 g/L PTAD的乙腈溶液,经0.22 μm滤膜过滤后,LC-MS/MS分析。通过与混合标准溶液比较,采用不同固相萃取柱时的回收率如图4所示。结果表明,C_18_ SPE柱对25-羟基维生素D回收率最低。相较于Silica SPE柱,HLB SPE柱对目标化合物具有更高的回收率,回收率范围为74.8%~92.7%。同时为避免Silica SPE柱使用过多有机试剂,本实验选择HLB SPE柱作为净化材料。

**图4:**
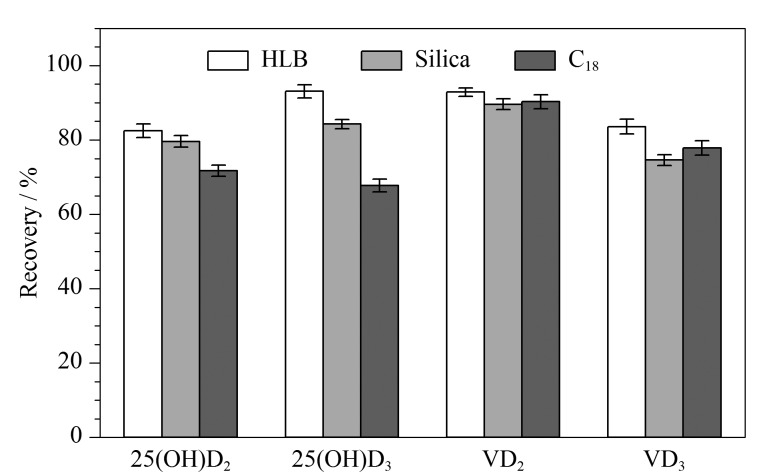
不同固相萃取柱对维生素D和25-羟基维生素D回收率的影响(*n*=3)

### 2.4 流动相的优化

在GB 5009.296-2023第三法中,流动相条件为在水-甲醇体系中添加甲酸和甲酸铵进行梯度洗脱^[16]^。本研究考察了在衍生条件下,3种不同流动相体系对目标化合物(质量浓度为10 μg/L)信噪比(*S/N*)的影响;同时按照国标方法测定非衍生条件下目标化合物(质量浓度为10 μg/L)的信噪比。3种不同流动相体系分别为流动相1: A相为0.05%甲酸-5 mmol/L甲酸铵水溶液,B相为0.05%甲酸-5 mmol/L甲酸铵甲醇溶液;流动相2: A相为0.1%甲酸水溶液,B相为0.1%甲酸甲醇溶液;流动相3: A相为0.1%甲酸-5 mmol/L甲胺水溶液,B相为0.1%甲酸-5 mmol/L甲胺甲醇溶液。不同流动相对8种目标化合物信噪比的影响见表2。

**表2:** 不同流动相体系对8种化合物信噪比的影响(*n*=3)

Compound	Non-derivatized/10^2^ (GB method^*^)	Derivatized/10^2^			
		MP1	MP2	MP3	
25(OH)D_2_	1.60	4.94	13.09	128.7	
25(OH)D_2_-d_6_	1.85	11.05	40.01	130.1	
25(OH)D_3_	2.32	8.08	14.76	64.70	
25(OH)D_3_-d_6_	2.49	5.41	10.34	44.43	
VD_2_	1.01	17.39	28.30	60.01	
VD_2_-d_3_	0.46	17.06	21.84	44.29	
VD_3_	1.06	27.94	26.82	54.56	
VD_3_-d_3_	0.84	22.63	23.27	60.29	

* GB 5009.296-2023 (the third method). MP: mobile phase; MP1: 0.05% formic acid-5 mmol/L ammonium formate in water and 0.05% formic acid-5 mmol/L ammonium formate in methanol; MP2: 0.1% formic acid in water and 0.1% formic acid in methanol; MP3: 0.1% formic acid-5 mmol/L methylamine in water and 0.1% formic acid-5 mmol/L methylamine in methanol.

结果表明,目标化合物衍生化处理后,信噪比均有明显提高,可见衍生化反应后电离效率得到提高,信号响应得到增强。在流动相中添加5 mmol/L甲胺,使目标化合物形成甲胺加合离子[M+CH_3_NH_3_]^+^,可进一步提升其信噪比。在含有甲胺的流动相条件下,25-羟基维生素D的信噪比明显提升。在甲胺环境下,PTAD与VD_3_衍生产物的二级质谱图如图5所示。本实验选择在水-甲醇体系中添加甲酸和甲胺,流动相为0.1%甲酸-5 mmol/L甲胺水溶液和0.1%甲酸-5 mmol/L甲胺甲醇溶液。

**图5:**
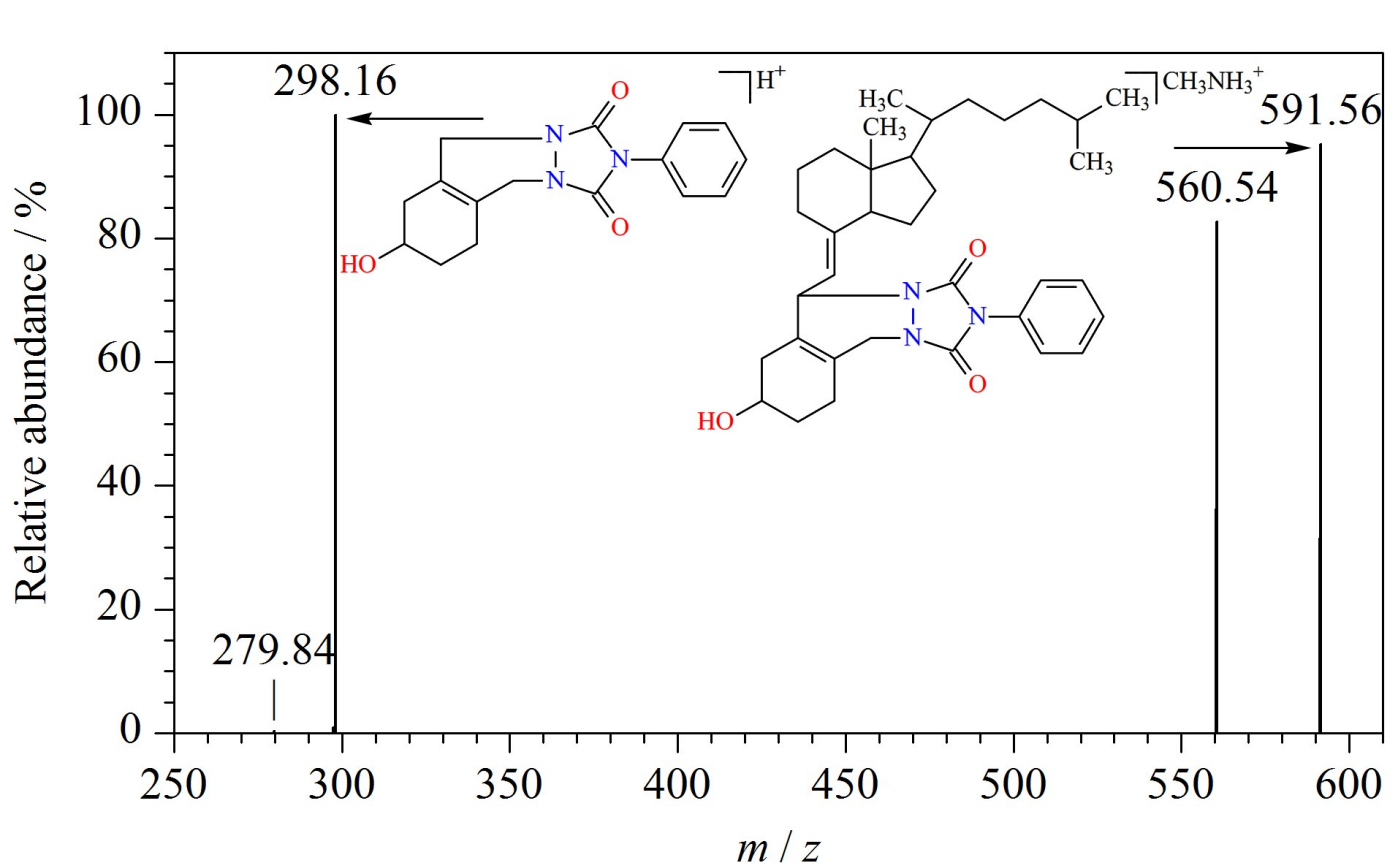
VD_3_经PTAD衍生后的二级质谱图(流动相中添加甲胺)

### 2.5 方法验证

#### 2.5.1 线性关系、检出限和定量限

在优化后的色谱和质谱检测条件下,将1.2节配制的系列标准工作溶液进行衍生化反应后,绘制标准曲线。以目标化合物的质量浓度与相应同位素内标的质量浓度比值为横坐标,以目标化合物与相应同位素内标的峰面积比值为纵坐标,确定标准曲线及线性范围内的相关系数。质量浓度为1.0 μg/L的维生素D及25-羟基维生素D混合标准溶液经PTAD衍生后的MRM色谱图见图6a。当取样量为5.00 g时,在羊肉样品中添加混合同位素内标,计算样品中维生素D及25-羟基维生素D含量,分别以3倍和10倍信噪比来确定方法的检出限和定量限。羊肉样品中维生素D及25-羟基维生素D衍生后的MRM色谱图见图6b。

**图6:**
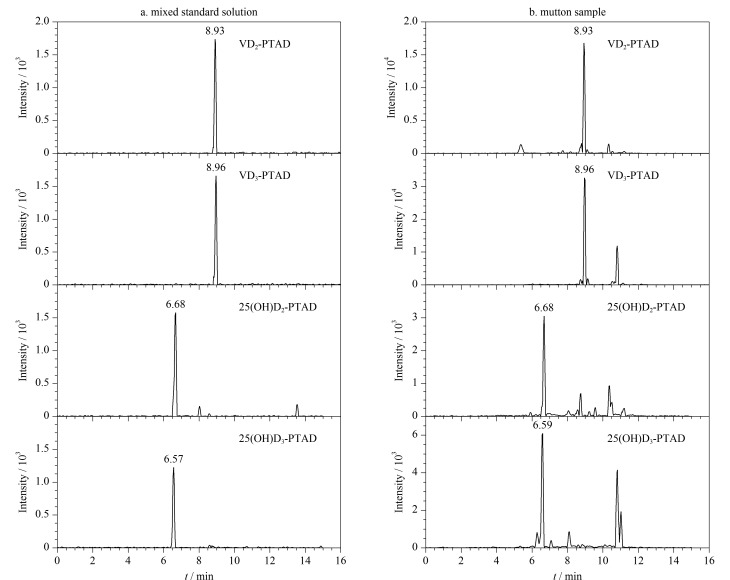
(a)维生素D和25-羟基维生素D混合标准溶液(1.0 μg/L)及(b)羊肉样品经PTAD衍生后的MRM色谱图

本方法中维生素D和25-羟基维生素D的线性范围、线性方程、相关系数、内标使用量、检出限和定量限见表3,同时与GB 5009.296-2023第三法进行了比较。结果表明:维生素D和25-羟基维生素D在0.2~50 μg/L范围内线性良好,相关系数(*r*^2^)≥0.9995。当固体试样取样量为5.00 g、定容1.00 mL时,维生素D及25-羟基维生素D的方法检出限(*S/N*=3)为0.018~0.066 μg/kg,方法定量限(*S/N*=10)为0.06~0.22 μg/kg。本方法测定维生素D及25-羟基维生素D的线性范围宽,较国标方法灵敏度显著提高,同时降低了氘代内标的使用量,有效降低了实验成本。本方法中维生素D与25-羟基维生素D的定量限为0.06~0.22 μg/kg,显著低于GB 5009.296-2023第三法中维生素D的定量限(5~10 μg/kg)。

**表3:** 维生素D和25-羟基维生素D的线性方程、相关系数、检出限、定量限和内标使用量

Compound	Linear equation	r^2^	LODs/(μg/kg)			LOQs/(μg/kg)			Dosages of IS/ng		
			This method	GB method^*^		This method	GB method^*^		This method	GB method^*^	
25(OH)D_2_	Y=1.0318X-0.0766	0.9997	0.027	/		0.09	/		5		/
25(OH)D_3_	Y=0.9692X-0.0749	0.9997	0.066	/		0.22	/		5		/
VD_2_	Y=1.6005X+0.0075	0.9995	0.024	3		0.08	10		5		200
VD_3_	Y=0.9460X+0.0108	0.9999	0.018	1.5		0.06	5		5		100

Linear range: 0.2-50 μg/L; *Y*: peak area ratio of analyte to IS; *X*: mass concentration ratio of analyte to IS; * GB 5009.296-2023 (the third method); /: not given.

#### 2.5.2 加标回收率和精密度

以脱脂乳粉为空白样品基质,添加低(0.20 μg/kg)、中(1.0 μg/kg)、高(5.0 μg/kg)3个水平的维生素D和25-羟基维生素D混合标准溶液,按本文所述方法进行提取、净化和测定,每个添加水平重复6次,考察方法的回收率和精密度。结果如表4所示,方法的平均加标回收率为92.6%~99.4%,相对标准偏差为3.6%~6.2%,表明该方法的加标回收率和精密度良好。

**表4:** 维生素D和25-羟基维生素D在脱脂乳粉中的回收率和精密度(*n*=6)

Compound	0.20 μg/kg			1.0 μg/kg			5.0 μg/kg		
	Rec./ %	RSD/ %		Rec./ %	RSD/ %		Rec./ %	RSD/ %	
25(OH)D_2_	93.6	4.6		96.8	5.3		98.4	3.7	
25(OH)D_3_	94.1	5.1		97.5	4.9		97.2	5.0	
VD_2_	94.4	4.7		98.5	6.2		99.3	4.1	
VD_3_	92.6	5.2		99.4	5.8		96.5	3.6	

Rec.: recovery.

#### 2.5.3 样品的基质效应

基质效应是由于离子化过程中样品中杂质组分与目标化合物相互竞争所引起的。本研究按照公式ME=(空白基质匹配标准曲线的斜率/纯溶剂标准曲线的斜率-1)×100%测定基质效应。分别测定以脱脂乳粉作为空白样品基质配制标准曲线和乙腈作为纯溶剂配制的标准曲线的斜率,不同净化条件下,维生素D和25-羟基维生素D的基质效应如图7所示。结果表明,25-羟基维生素D在脱脂乳粉样品基质中呈现基质抑制效应,维生素D在样品基质中呈现基质增强效应。未经净化处理条件下,维生素D和25-羟基维生素D的ME范围为-54.5%~94.0%。通过固相萃取柱净化后,25-羟基维生素D的ME值为-21.2%~-38.3%,维生素D的ME值为53.3%~68.0%,说明净化处理降低了基质效应的影响。

**图7:**
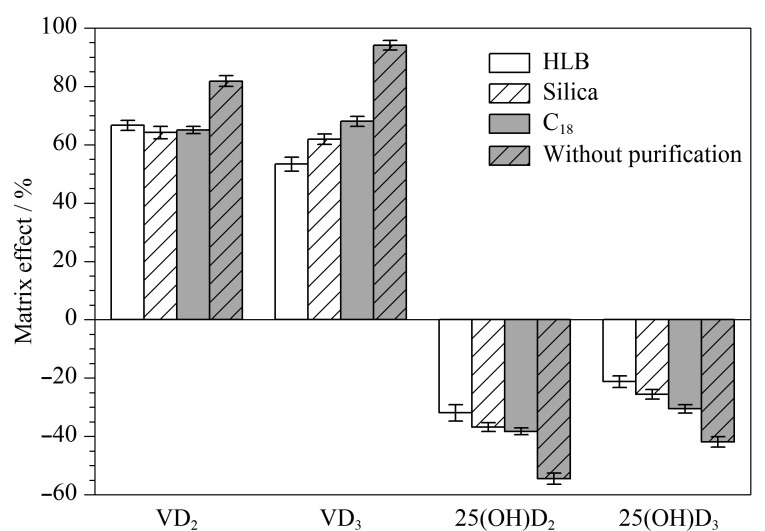
维生素D和25-羟基维生素D在空白样品中的ME(*n*=3)

本实验通过同位素内标法抵消基质效应,通过内标法测定维生素D和25-羟基维生素D在脱脂乳粉中标准曲线的斜率,再次评估绝对基质效应,维生素D和25-羟基维生素D的ME绝对值在1.81%~16.9%范围内,说明其基质效应得到了有效补偿。

### 2.6 实际样品的检测

应用本研究建立的方法对市售不同动物源性食品中维生素D及25-羟基维生素D的含量进行检测,每种样品取3组平行样检测,进样质量浓度和样品含量见表5。

**表5:** 食品中维生素D及25-羟基维生素D的含量测定(*n*=3)

Sample	Sample injection mass concentrations/(μg/L)					Contents/(μg/kg)						
	25(OH)D_2_	25(OH)D_3_	VD_2_	VD_3_		25(OH)D_2_	25(OH)D_3_		VD_2_		VD_3_	
Nonfat milk powder	-	-	-	-		-		-		-		-
Flesh of fish	-	-	6.101	37.65		-		-		1.22		7.53
Flesh of shrimp	-	1.965	-	-		-		0.393		-		-
Pork meat	-	5.851	-	14.65		-		1.17		-		2.93
Mutton	4.155	7.202	6.950	6.650		0.831		1.44		1.39		1.33
Egg yolk	-	34.50	-	40.50		-		13.8		-		16.2
Egg white	-	4.315	-	3.365		-		0.0863		-		0.0673
Duck blood	-	45.50	-	5.103		-		18.2		-		2.04

-: not detected

由表5可以看出,鸡蛋黄中VD_3_含量最高,含量为16.2 μg/kg,鸭血中25(OH)D_3_含量最高,含量为18.2 μg/kg。

## 3 结论

本研究使用PTAD作为衍生试剂,对衍生化条件、样品前处理条件、色谱分离条件和质谱检测条件进行了优化,建立了衍生化-超高效液相色谱-串联质谱同时测定动物源性食品中4种D族维生素物质的方法。本方法有效降低了样品基质干扰,灵敏度高,定量准确,重复性好,可满足动物源性食品中D族维生素物质的测定要求。
